# On data sharing in computational drug discovery and the need for data notes

**DOI:** 10.12688/f1000research.5742.1

**Published:** 2014-11-14

**Authors:** Jürgen Bajorath

**Affiliations:** 1 Department of Life Science Informatics, B-IT, LIMES Program Unit Chemical Biology and Medicinal Chemistry, Rheinische Friedrich-Wilhelms-Universität, Dahlmannstr, Bonn, D-53113, Germany

## Abstract

In the big data era, the scientific community is in need of better practices and infrastructures for data deposition and sharing. In addition, scientific journals are challenged with formulating, implementing, and enforcing commonly accepted data deposition guidelines and addressing problems associated with the use of proprietary data. Furthermore, new publication formats are required to specifically focus on data, their organization, and related issues and raise awareness of data heterogeneity and complexity. Such types of publications should also present a forum for evaluating and discussing specifics of data upon which follow-up investigations are based. Data articles/notes introduced by *F1000Research* represent an important step in the right direction.

## Big data

In the life sciences and pharmaceutical research, data volumes grow at unprecedented rates, which presents new challenges for data handling, analysis, and knowledge extraction ^1, 2^. Not only mere data volumes complicate matters, but also increasing data heterogeneity and complexity ^2^, which affect data curation as well as database design and maintenance. In addition, new computational methodologies are required for effective data mining. In the big data era, there is generally increasing awareness of data-related issues including accessibility and sharing, which are commented on in the following.

## Data sharing

This editorial considers data sharing and communication practices from a chemistry-centric computational drug discovery perspective ^2^, an area in which data sharing is increasingly discussed ^3^, and with a particular focus on scientific publication standards. Although this perspective is narrow, at least some of the conclusions drawn are probably of general relevance.

Small molecule-associated data with relevance for drug discovery include, first and foremost, chemical structure, compound activity, biological screening, and pharmacological data, which rapidly grow. Discovery-relevant data are produced in both pharmaceutical and academic research environments and often kept proprietary, for obvious reasons. This is not only the case in the pharmaceutical industry, as academic drug discovery centers are on the rise in many places (see, for example http://addconsortium.org/). The propriety nature of drug discovery data principally hinders data sharing. However, new (and at least in part) publically funded initiatives are established that integrate commercial and academic drug discovery-related research and foster data exchange (see, for example, http://www.openphacts.org/). In addition to such initiatives, scientific publications provide, in principle, a major platform for data sharing.

## Publication standards

The big data era provides ample opportunities for computational analysis and design. Large-scale data mining studies are increasingly reported that attempt to derive knowledge from discovery-relevant data concerning, for example, preferred drug properties, polypharmacology, or attractive drug targets. In addition, computational models are derived on the basis of available data, for example, to predict novel active compounds or drug side effects, resulting in many computational publications. In the latter case, different from data mining efforts, large data volumes are often not essential for the derivation of predictive computational models. Rather, data sets of limited size (comprising, for example, active and inactive compounds) are often sufficient for machine learning. Thus, in these cases, data requirements and challenges have not substantially changed in recent years.

Similar to any experimental study for which reagents should be made freely available to ensure reproducibility, computational publications should provide the data upon which an analysis or prediction is based. Such requirements reflect basic scientific publication standards. Journals typically formulate deposition requirements for data (and other materials) in their guidelines, but these deposition requirements are often not rigorously enforced, especially in the case of computational studies using proprietary or in-house curated (public) data. Here, journals are challenged to synchronize their efforts, establish transparent and generally applicable data deposition criteria – and rigorously enforce them.

Most computational modeling efforts should be readily possible on the basis of large amounts of currently available compound and/or target data. If models are built, or methods developed, on the basis of proprietary data, the data should be made available – or the models/methods, if tailored towards proprietary data, should not be published. For data mining efforts, exceptions might occasionally be made if knowledge derived from proprietary data could not possibly be obtained on the basis of public domain data (which might apply, for instance, to fundamental analyses of drug discovery data accumulated in the pharmaceutical industry over time). In such cases, a waiver on data deposition requirements might be considered, if it is in the best interest of the scientific community (an option offered, for example, by the *Journal of Medicinal Chemistry*).

For computational publications, an essentially still open question is whether or not executable versions or source code of in-house generated computer programs used for a particular analysis should be provided when a study is published. This also includes implementations of newly developed algorithms or computational methods. This question is often viewed controversially and not specifically addressed in author guidelines. One might argue that executable versions (but not necessarily source code) should be regarded as “reagents” and fall under data deposition requirements. This would then also apply to computational studies reported by software companies. On the other hand, if sufficient details are provided in a publication to fully re-implement a new computational methodology, reproducibility can also be ensured (an option subjectively favored by the author). However, studies using proprietary algorithms that are not disclosed (for example, algorithms developed by software companies) should not merit publication, similar to the use of proprietary data for modeling. Computational journals will also be required to take a clear stand on such software-related issues, which is currently not necessarily the case, and consistently apply criteria put in place.

## New publication formats

In light of increasing data volumes, complexity, and heterogeneity, new publication formats that primarily focus on source data should be very helpful to the scientific community (in any field). In scientific publications utilizing data sets, there often is insufficient room to describe the data and their organization in sufficient detail to emphasize data-related issues that are not a primary focal point of the investigation. This often represents a shortcoming of computational publications and limits the general utility of computational methods (especially when specifically curated data are used to establish them). From this viewpoint, the introduction of data articles/notes by *F1000Research* is considered a very valuable addition to the scientific publication landscape. By design, data articles support rigorous data deposition, provided they can be linked to open access infrastructures. For *F1000Research*’s data notes, this has been accomplished by directly depositing reported data via freely accessible stable repositories, including CERN’s ZENODO platform ( http://www.zenodo.org/), hence providing an efficient and informative solution. Moreover, data notes provide a prime publication format for organizing and reporting data sets that have been generated over time in research environments, often for different purposes and in the context of different studies. Once published, an article reporting such data sets represents a generally accessible reference, hence alleviating the need to respond to and instruct interested investigators on a case-by-case basis (which is laborious and not necessarily consistent). Reference 4 represents an exemplary data note that specifies many data sets and software tools for computational drug discovery that have originated from our research group and are made freely available as ZENODO depositions. In combination with the corresponding data note, which is essential for educated data use, such open access depositions provide a meaningful alternative to maintaining local websites for data/tool distribution.

Hence, there are multiple reasons to support data notes and related publication formats in different journals. For data notes, an added bonus of the open peer review and discussion culture promoted by *F1000Research* is that it provides a forum for addressing individual data-related questions or comments in a way accessible to a wide audience. This is expected to further increase the utility of open access data depositions.
